# The perceived impact of the National Health Service on personalised nutrition service delivery among the UK public

**DOI:** 10.1017/S0007114515000045

**Published:** 2015-03-27

**Authors:** Rosalind Fallaize, Anna L. Macready, Laurie T. Butler, Judi A. Ellis, Aleksandra Berezowska, Arnout R. H. Fischer, Marianne C. Walsh, Caroline Gallagher, Barbara J. Stewart-Knox, Sharon Kuznesof, Lynn J. Frewer, Mike J. Gibney, Julie A. Lovegrove

**Affiliations:** 1 Hugh Sinclair Unit of Human Nutrition and Institute for Cardiovascular and Metabolic Research, University of Reading, Whiteknights, ReadingRG6 6AP, UK; 2 Department of Psychology, University of Reading, Earley Gate, Whiteknights, ReadingRG6 6AL, UK; 3 Marketing and Consumer Behaviour Group, Wageningen University, Wageningen, PO Box 8130, The Netherlands; 4 UCD Institute of Food and Health, UCD Centre for Molecular Innovation, Science Centre South, University College Dublin, Belfield, Dublin 4, Republic of Ireland; 5 Faculty of Social and International Studies, University of Bradford, BradfordBD7 1DP, UK; 6 School of Agriculture, Food and Rural Development, Agriculture Building, University of Newcastle, Newcastle upon TyneNE1 7RU, UK

**Keywords:** Personalised nutrition, National Health Service, Disease prevention, Nutrigenomics, Focus groups

## Abstract

Personalised nutrition (PN) has the potential to reduce disease risk and optimise health and performance. Although previous research has shown good acceptance of the concept of PN in the UK, preferences regarding the delivery of a PN service (e.g. online *v.* face-to-face) are not fully understood. It is anticipated that the presence of a free at point of delivery healthcare system, the National Health Service (NHS), in the UK may have an impact on end-user preferences for deliverances. To determine this, supplementary analysis of qualitative data obtained from focus group discussions on PN service delivery, collected as part of the Food4Me project in the UK and Ireland, was undertaken. Irish data provided comparative analysis of a healthcare system that is not provided free of charge at the point of delivery to the entire population. Analyses were conducted using the ‘framework approach’ described by Rabiee (Focus-group interview and data analysis. *Proc Nutr Soc* 63, 655-660). There was a preference for services to be led by the government and delivered face-to-face, which was perceived to increase trust and transparency, and add value. Both countries associated paying for nutritional advice with increased commitment and motivation to follow guidelines. Contrary to Ireland, however, and despite the perceived benefit of paying, UK discussants still expected PN services to be delivered free of charge by the NHS. Consideration of this unique challenge of free healthcare that is embedded in the NHS culture will be crucial when introducing PN to the UK.

In 2011, prevention of non-communicable disease (NCD) was declared a global priority by the UN, and governments were tasked with a 25 % reduction in premature NCD mortality by 2025^(^ ^1^ ^)^. The risk factors to be targeted include unhealthy diets and physical inactivity, which are known to account for up to 80 % of NCD^(^ ^2^ ^)^. It is estimated that population-wide behavioural changes are more beneficial in reducing chronic disease risk than drug- and hospital-based interventions^(^ ^3^ ^)^; thus, the UN have recommended a multi-sectorial response that goes beyond the current healthcare provision. Furthermore, modelling studies have predicted significant cost savings using population-wide prevention programmes^(^ ^4^ ^)^.

Tailoring information to make it personally relevant is one of the key steps for improving health information delivery^(^ ^5^ ^)^ and facilitating behaviour change. As such, health promotion messages that aim only to increase public knowledge are ineffective^(^ ^6^ ^)^. The evolution of personalised nutrition (PN), from tailoring diets based on eating behaviours and anthropometrics to phenotypic characteristics and genetic variations, is anticipated to change this and thereby improve the dietary prevention of NCD. Nutrigenomic-based PN has the potential to reduce disease risk, optimise health and performance, and assist in the treatment of chronic disease^(^ ^7^ ^–^ ^13^ ^)^. Moving away from a ‘one-size-fits-all’ approach to personalisation of health care is already a priority in the medical treatment of several conditions including mental health, arthritis and cancer^(^ ^14^ ^–^ ^16^ ^)^. While nutrigenomic-based PN advice is found to be beneficial in the treatment of obesity, its impact on the prevention of NCD is still inconclusive^(^ ^17^ ^,^ ^18^ ^)^.

If nutrigenomic-based PN is proven to be effective, it will be necessary to ensure that the public would be willing to engage with a PN service^(^ ^8^ ^)^. Although users tend to look at the Internet first when searching for disease-specific information, they place greater trust in information provided face-to-face by healthcare professionals (HCP)^(^ ^19^ ^)^. However, Interactive Health Communication technology, that uses computer applications and Internet services, may lead to lower costs of nutrition intervention^(^ ^20^ ^)^, and is more likely to be read and remembered compared with standard guidelines^(^ ^21^ ^)^. As such, online healthcare provision is becoming more common alongside the transition from HCP-led care to individual management of health^(^ ^22^ ^,^ ^23^ ^)^. Previous research in Europe has suggested a preference for one-to-one communication of gene-based PN advice by a HCP^(^ ^24^ ^–^ ^26^ ^)^, although this business model does not exist currently^(^ ^27^ ^)^. Before such services being introduced in the UK as a means of preventing NCD, it is important to identify the best route of entry for PN delivery: would users be happy to use online companies such as 23andme^(^ ^28^ ^)^ or would they prefer talking with a HCP face-to-face? General health care is provided free of charge at the point of delivery in the UK via the National Health Service (NHS), and it is anticipated that this may have an impact on preferences for the delivery of PN. Direct comparison with a country, such as Ireland, where health care is no longer freely available to the entire population may provide further insights into the impact of the NHS on the preference for delivery.

Focus group interviews are a tried and tested methodology for the exploration, planning and evaluation of health promotion and nutrition interventions^(^ ^29^ ^)^. In addition, they can be beneficial for planning care management and strategy development^(^ ^30^ ^)^ and may, therefore, assist in the development of novel methods of PN delivery.

In order to determine the impact of the NHS on PN delivery and public preferences, qualitative data relating to PN service delivery in the UK and Ireland were generated through supplementary analysis^(^ ^31^ ^)^ of focus group data collected as part of the European Union Framework Seven project, Food4Me^(^ ^32^ ^)^ (http://www.food4me.org). The aims of this analysis are to explore UK and Irish participants' preference for PN service delivery and to contrast this opinion between the two European member states. The present study provides an in-depth analysis of the relationship between service provider and differences in national healthcare provision that was briefly identified in the primary studies^(^ ^33^ ^,^ ^34^ ^)^.

## Methods

Ethical approval for the present study was granted from the School of Psychology and Clinical Language Sciences, University of Reading, UK, and the Human Research Ethics Committee, University College Dublin, Ireland.

### Participants

A total of eight focus groups were conducted, four at each site (Reading, UK, and Dublin, Ireland). In total, seventy-three participants were recruited using local social research agencies. Recruitment was conducted in line with the Market Research Society Ethical Code of Conduct^(^ ^35^ ^)^. Each focus group session consisted of eight to ten discussants with an approximately equal sex split. Discussants were stratified according to age profile (‘mixed age adults’, 18–65 years; or ‘older adults’, 30–65 years) in order to enable the identification of age-specific issues. Each age profile was represented in two theme-guided discussions, as described below.

Volunteers were free-living and self-reported as healthy as determined by a screening health questionnaire. Exclusion criteria included the following: presence of a learning disability or ailment that may impair ability to participate and communicate; specialist knowledge in the area of food, diet, health or genomics; current or previous participation in clinical nutrition trials; participation in focus groups relating to PN; having previously taken part in focus group discussions. Volunteers were required to speak English fluently.

### Materials and focus group discussion guides

Two focus group discussion guides were used in the present study. The first group, ‘Consumer Perceptions of PN’ (CPPN), included questions on the knowledge and understanding of PN, service delivery of PN and associated ethical, legal and social issues. The CPPN scripts introduced three PN service scenarios that required increasing levels of personal information. Scenario 1 was based on the provision of lifestyle-related data, for which discussants were asked to ‘imagine and comment on a scenario where they wanted to change their diet to improve their health through an online provider’^(^ ^33^ ^)^. In scenario 2, participants were asked to consider providing phenotypic information via a home test kit (finger prick blood test, waist and hip circumference), and in scenario 3, they were asked about providing genetic information via a home cheek swab kit (see Stewart-Knox *et al.* ^(^ ^33^ ^)^ for detailed methods).

The second discussion guide script, ‘PN business models’, aimed to investigate opinions towards the nine PN business model archetypes proposed by Ronteltap *et al.* ^(^ ^27^ ^)^, and was facilitated using fictitious flyers representing these business models. Business models differed according to information required (dietary intake, phenotype and genotype), service provider (dietitian, company and government/employer), advice background (scientific, alternative medicine and success stories), costing plan, feedback type and frequency, and delivery method (online, mail and personal contact) (see Berezowska *et al.* ^(^ ^34^ ^)^ for detailed methods).

### Focus group procedure

Focus groups were conducted between October and December 2011 using standardised semi-structured discussion protocols with prompts. Before each session, volunteers signed an informed consent form. A moderator and an observer were present at each session and discussions were audio-recorded with permission. Each session lasted between 75 and 150 min, including a 10-min refreshment break halfway through the session. To maintain consistency in data collection between centres, a 2-d training workshop for moderators was held 1 month before the focus groups were conducted.

### Data analysis

Data were transcribed verbatim and verified by an independent researcher. Manual analyses of content relating to PN delivery were carried out using the ‘Framework’ approach described by Rabiee^(^ ^30^ ^)^. Two independent researchers conducted the analyses, following five key stages: familiarisation; identification of a thematic framework; indexing; charting; mapping; interpretation. The aim of familiarisation, achieved by going through tape recordings, transcripts and observation notes, was to get a sense of the focus group data as a whole. Identification of a thematic framework involved forming short phrases, ideas and concepts arising from the data starting to develop themes/categories. The third stage, indexing, involved sorting quotes and making comparisons. Quotes were then re-sorted under the identified themes in the fourth stage, charting. During this latter stage, data were also further reduced into ‘relevant’ quotes. Mapping and interpretation, the final stage of the ‘Framework’ approach, involved the interpretation of data identifying links and relationships. Where relevant, minority opinions or noteworthy ideas relating to themes were included in the results, although these were clearly marked as alternative views.

## Results

### Participant characteristics

The sex-specific mean ages of the focus group discussants are summarised in Table 1 (CPPN) and Table 2 (business models). All participants were in socio-economic group B (middle class: intermediate managerial, administrative or professional) or C1 (lower middle class: supervisory or clerical, junior managerial, administrative or professional).

**Table 1:** Participant characteristics of UK and Irish consumer perceptions focus group discussants

	UK	Ireland
	Group 1 (*n* 10)*	Group 2 (*n* 10)†	Group 1 (*n* 8)*	Group 2 (*n* 8)†
Age (%)				
18–30 years	40·0	0·0	12·5	0·0
30–45 years	50·0	50·0	37·5	50·0
45–65 years	10·0	50·0	50·0	37·5
Not recorded	0·0	0·0	0·0	12·5
Sex (%)				
Male	50·0	50·0	37·5	37·5
Female	50·0	50·0	62·5	62·5
Not recorded	0·0	0·0	0·0	0·0
Marital status (%)				
Married	50·0	90·0	37·5	75·0
Lives with partner	0·0	0·0	0·0	12·5
Divorced	0·0	10·0	0·0	0·0
Single	50·0	0·0	62·5	12·5
Not reported	0·0	0·0	0·0	0·0

* 18–65 years.

† 30–65 years.

**Table 2:** Participant characteristics of UK and Irish business models focus group discussants

	UK	Ireland
	Group 3 (*n* 9)*	Group 4 (*n* 8)†	Group 3 (*n* 10)*	Group 4 (*n* 10)†
Age (%)				
18–30 years	22·2	0·0	30·0	0·0
30–45 years	66·7	50·0	50·0	80·0
45–65 years	11·1	50·0	20·0	20·0
Not recorded	0·0	0·0	0·0	0·0
Sex (%)				
Male	44·4	37·5	50·0	40·0
Female	55·6	62·5	50·0	60·0
Not recorded	0·0	0·0	0·0	0·0
Marital status (%)				
Married	44·4	50·0	20·0	50·0
Lives with partner	22·2	25·0	20·0	20·0
Divorced	0·0	12·5	0·0	0·0
Single	33·3	12·5	60·0	30·0
Not reported	0·0	0·0	0·0	0·0

* 18–65 years.

† 30–65 years.

**Fig. 1:**
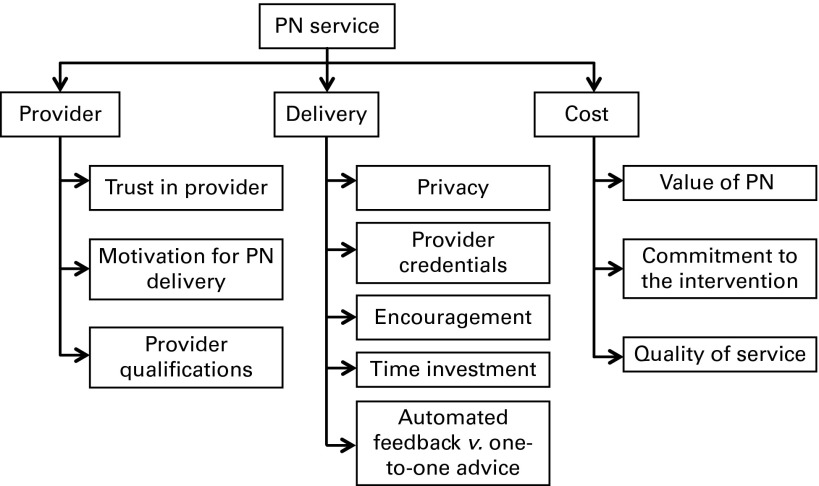
Themes emerging from supplementary framework analysis of personalised nutrition (PN) service using Food4Me focus groups (UK and Ireland).

Themes relating to PN service offerings were separated into three categories: service provider; delivery; cost; that is, who is providing the PN service, how and where it is being provided, and what the expected cost of the service might be (see Fig. 1). Suggested alternatives and preferences are also presented.

### Personalised nutrition service provider

Themes relating to PN service provider were present in all focus groups. Providers appraised by UK and Irish discussants included supermarkets, pharmacies, health services (both public, e.g. the NHS, and private, e.g. BUPA) and online companies.

#### Motivation for personalised nutrition delivery

Discussants were concerned that commercial companies may be motivated by sales as opposed to improving health outcomes. However, the NHS was seen as ‘independent’ and interested in the individual's health, not personal gain:

‘You know a lot of websites that I wouldn't recognise the name I'd just assume in the end that they were going to try and sell me a diet supplement or something […]’ (Ireland)

‘… If it's done for, for the NHS then […] it's probably a bit more appealing to people to use, because I know, they are independent and they are interested in you.’ (UK)

However, government-run services were not always seen as positive; when discussing a workplace PN service run by the government, one UK discussant said:

‘[…] I felt that it was more to do with getting statistics about a healthy lifestyle and increasing employee productivity than it was genuinely about, you know, a tailored diet plan for an individual …’ (UK)

#### Trust in provider

‘Well-known’ and ‘reputable’ providers such as BUPA (private healthcare provider) generated greater trust on the part of discussants than more anonymous Web providers. Concerns were raised regarding the transportation and storage of personal data (e.g. blood and gene results), and the advice that commercial companies might provide based on their data. Government backing or support for a PN scheme was considered to be largely positive, providing validation and engendered trust:

‘I suppose we are all just wary of security and not knowing maybe the company behind. a website, how reputable they are, and whether the samples are going to get mixed up or fall into the wrong hands …’ (Ireland)

‘…I would trust it more if they say, oh, we are part of, like a, you know, government funded project, I would trust it more.’ (UK)

However, government involvement was seen as intrusive and representing a ‘Big Brother’ approach in the workplace; ‘Big Brother’ is a term used to describe a person or an organisation that exercises dictatorial control over people's lives, including surveillance. This opinion was held by discussants in both countries:

‘Well I think though if the government, if it said the government was running an initiative and you just had to sign up and say to a website or something I would accept that bit if they took the work thing out, I think it's just too Big Brother, the government and your work.’ (Ireland)

‘It's very Big Brother is watching you’. (UK)

#### Provider qualifications

Provider of PN was discussed in terms of both a company and an individual. An Irish discussant noted that in order to sign up to an online PN service:

‘… you need to have a professional on board as well, rather than just an online service.’ (Ireland)

Discussants generally agreed that for engagement in PN, it was important for dietitians, nutritionists or even their general practitioners (GP) to have the proper credentials and qualifications to provide dietary advice:

‘I'd hope the GP would have done a few courses outside of just the normal medical profession ones, so if he had actually attended a course on nutrition or something like that …’ (Ireland)

‘I think I would like to have more about the credentials of the person doing the assessment, like actual confirmation as to their qualifications […] I'd like to know that they have the relevant qualifications and that they are somebody with that knowledge that they can empower to you for whatever your specific requirements are’ (Ireland).

#### Preference

Despite the variance in expectation of service provider, there was a preference for all PN services to be delivered by the NHS in the UK. The NHS was seen as more credible, trustworthy and confidential than commercial/private companies:

‘… unless it was with a doctor I would not be giving a cheek swab because […] you've got the confidentiality with the medical profession which you don't have with the, a private corporation, I wouldn't be willing to do that.’ (UK)

‘… BUPA or NHS, I don't, me personally, I don't think I'd go with anyone else. I don't want to go to Tesco's or you know Waitrose or whoever, I'd want to go just to them.’ (UK)

Irish discussants also expressed a preference for PN services offered by medical centres and backed by their Department of Health:

‘I'm more suspicious of the Internet, I mean if it was a local company or a hospital you went down to and they had a genetic centre and you could get these services done I might be very interested …’ (Ireland)

#### Alternative view

While the NHS was a preferred source of PN, UK discussants noted that it was unlikely that they could afford to provide DNA testing and that referral from a GP to a private health institution could be a viable alternative:

‘So if you go to your GP and my GP says, “well look you're healthy and the NHS is just not going to cover this but you can go through BUPA and they can analyse it, they'll take some DNA and everything”. Then I would be much more comfortable and I would think okay, it's my GP via BUPA.’ (UK)

### Delivery of personalised nutrition service

Over the course of the focus groups, discussants appraised face-to-face, Internet and telephone delivery of PN advice.

#### Privacy

Respondents felt that it was easier to trust providers with their private information (e.g. credit card details and genetic results) when PN was delivered ‘face-to-face’:

‘Yes, like face-to-face would be better because […] it's just that there's nothing more valuable than your credit card information or your genetic information so no, not on emails.’ (Ireland)

Seeing someone in person also gave them the opportunity to validate the providers' personal characteristics, including their health, which if positive, was associated with increased trust in that individual:

‘I think personal appearance is also important because I wouldn't trust somebody who says to me that I should go away and eat this, this and this and you can see that the person is not healthy at all …’ (Ireland)

#### Provider credentials

In both countries, benefits were seen with Web-based services in providing anonymity to the user. However, unaccredited online providers were a concern for delivery of PN services:

‘I would say with, um, um, online, er, websites and that, you don't know if it's one […] person sitting in their bedroom running it’ (UK)

A benefit associated with face-to-face PN delivery is that it would enable discussants to check the provider's credentials using visual and other interpersonal cues, which increased trust in the service:

‘I would only trust if I met someone face to face that they were a professional dietitian because I can ask them for credentials, online I wouldn't believe them’ (Ireland)

#### Automated feedback v. one-to-one advice

Discussants appeared doubtful that a fully automated online service would provide the same amount of in-depth information as a one-to-one service:

‘I'd prefer to see a person than go on a website […] because they can see you and you can talk to them and you can, you can talk more than what you would if you were just putting information into a website.’ (Ireland)

In Ireland, face-to-face delivery was also seen as a more effective way for both participants and provider to monitor safety and efficacy:

‘Hmm also isn't there is a risk like a medical risk that if the diet doesn't suit you and you don't see anyone and you continue it and you have paid up for something that is obviously not good for you’ (Ireland)

#### Time investment

Concerns were raised regarding the amount of time it may take to fill in dietary questionnaires online and discussants commented that it might be easier for someone to go through their diet history over the telephone:

‘I think if I, I had it I'd like someone to call me, call me back and I'd rather speak to them over the phone about it rather, coz it could, it could take forever. To fill this information in and it's a lot quicker just to speak to somebody on the phone about it.’ (UK)

#### Encouragement

In addition, online delivery was seen to have less potential than face-to-face delivery for encouraging people to change their diet. The relationship with the dietary advice provider appeared to be dependent on body language, which was not visible online:

‘It's the motivation factor as well, that if you meet someone weekly you stay motivated to stay on your diet plan and exercise weekly then’ (Ireland)

#### Preferences

Overall, these factors led to a preference for PN delivery to be face-to-face:

‘I think if you're actually speaking to somebody, I dunno, for me I prefer that, to actually have somebody to sit with me and sort of go through everything that could be tailored and give you that encouragement.’ (UK)

‘I wouldn't do this at all. Hmm, you know if I, if I was sufficiently, had a strong enough motivation to have a need for all of this I'd, I'd want to see something, I'd want to see, hmm, you know I'd want to go to a place and see a person, I wouldn't get involved with a website.’ (Ireland)

#### Alternative view

In order to combine the preference for face-to-face delivery with an Internet-based approach, an Irish discussant suggested that Skype could be used:

‘There could be another choice though like Skype. You can see the doctor face to face but you don't have time to go to a nutritionist’ (Ireland)

### Cost of personalised nutrition service

Several themes emerged in relation to the cost of a PN service. These were as follows: value, commitment and the expected quality and detail of the feedback that would be provided.

#### Value of personalised nutrition service

Discussants questioned whether a PN service based on dietary information alone provided any added benefit than the nutritional information already freely available online, and were therefore unwilling to pay for it:

‘I wouldn't pay […] for someone just to look at what I eat and tell me what I should eat because I know that myself, I can look up that information myself …’ (UK)

When blood tests were involved, the cost could more easily be justified. In Ireland, participants agreed they would pay for a PN service as long as there was good value for money and the information provided went beyond public health guidelines. However, UK discussants felt that this service was already freely available from their GP surgery or hospital, so were less willing to pay:

‘You can go to a GP and have a cholesterol test, you can have blood pressure, you can have blood tests free of charge. So I'm not entirely convinced that there's too much added value from, from these kits.’ (UK)

‘… you wouldn't pay that for, I'd rather go and see a doctor of medicine who would refer me to a dietitian in the hospital, and get, get the same benefits.’ (UK)

In the UK, cost was seen as a barrier to PN uptake, as health services delivered by the NHS are provided free of charge:

‘I don't think I'd be willing to pay for it but I would be willing to use it, because I'm used to just getting everything medical free, because it's the culture in the UK…If I was, lived abroad where you didn't get, er, free national health care then, then yeah, I would expect to-to pay …’ (UK)

Conversely, Irish discussants did not expect a PN service to be provided free of charge and instead discussed the cost of the intervention in relation to existing healthcare costs. It was noted that the cost of a PN intervention could be covered using the insurance credits already used to access allied health professionals such as hygienists:

‘There is obviously a need for this kind of thing, hmm, so if this service would need to be very competitive for you, if you are already going to see somebody like that, hmm, I don't know, say see a doctor for 50–55 euros this would have to come in very keen, otherwise why are you going to do it.’ (Ireland)

‘It could be more, I mean hygienists get paid 80 euro so I say you would pay more to a nutritionist's.’ (Ireland)

#### Commitment to the intervention

Discussants in both countries perceived the act of paying for a PN service as symbolising commitment to that service:

‘When I thought about myself psychologically, if I get something for free […]. It has little value for me. If I pay out that amount of money and that level of commitment and that means that I'm committed, so the action of paying a lot of money […] means that I […] will commit. You know I'm not going to do that lightly, so even before I go there I am committed …’ (Ireland)

‘I think if you've paid that much money, then you're more likely to stick to it, because there's obviously a very good reason for you going there and paying that much money to do it, that you, you've got a good sound reason for doing it and a good motivation.’ (UK)

#### Quality of service

The cost of intervention was also linked to the quality and detail of the feedback with respondents expecting to pay a greater amount for more personalisation and vice versa. Higher costs were also associated with one-to-one services (including telephone contact) and professional providers:

‘… If you're paying £20 a month then you wouldn't really expect too much of a personal service: it would be like an email or a letter. But if you were paying hundreds a month as you were saying then you would definitely expect that one-to-one feedback …’ (UK)

‘… if I'm paying that amount of money, I want to have […] some kind of scientific tests being done in a professional, like, hospital, somewhere better than putting anything in the post or online.’ (UK)

Discussants in both the UK and Ireland noted that the cost of DNA testing was likely to be greater than that for PN based on dietary intake alone. Among those in Ireland, however, cost was not necessarily associated with quality:

‘I don't automatically associate cost of something with the quality of something so maybe that's a disassociation I have personally. If something is expensive doesn't necessary mean that it's good.’ (Ireland)

#### Preference

As noted, there was a preference for PN to be delivered free of charge in the UK, particularly when PN was based on just dietary intake and blood measures. Discussants commented that they would rather see their GP for free than pay for an online PN service. However, while it was expected that one-to-one services would cost more, this delivery method was still preferred:

‘I think I'd rather pay more to be able to go to a clinic than, than getting it cheap over the Internet.’ (UK)

## Discussion

The present supplementary analysis of data obtained from focus groups on behalf of the Food4Me study (http://www.food4me.org) aimed to explore the attitudes towards provider and delivery of PN in the UK and to contrast this opinion with that of Irish discussants. Existing research on willingness to undergo genetic testing for the prevention of NCD suggests that the UK public are largely accepting of this application of technology^(^ ^26^ ^,^ ^36^ ^)^. However, the preferred method of delivery of nutrigenomic-based PN is not fully understood. Results from the present study suggest that the presence of a free healthcare system, the NHS, may influence this preference. For example, while discussants in both countries preferred for PN to be led by government services, there was a clear difference in opinion with regards to paying for this service. UK discussants perceived that PN, especially when based on dietary intake and blood measures alone, should be provided free of charge by the NHS. Previous empirical research has also identified cost as a potential barrier towards uptake of nutrigenomic-based PN in the UK; in a survey of public interest towards personal genome tests, willingness to undergo testing dropped from 48 to 5 % when the personal genome test was associated with a fee of £250^(^ ^24^ ^)^. A core principle of the NHS is that ‘it be free at the point of delivery’^(^ ^37^ ^)^, whereas the majority of the Irish population are charged for appointments with a HCP. Therefore, Irish discussants appraised the cost of PN services in terms of these interventions, with no expectation of free provision. However, there was some doubt amongst UK discussants, that the NHS would be able to provide a free PN service aimed at the prevention of NCD. Referral by a public sector physician or HCP to a private healthcare system (e.g. BUPA) was seen as a viable alternative to direct provision by the NHS, which suggested that consumers are more trustful of physicians working for public healthcare companies compared with those working in private institutions.

Regardless of preference, discussants from both countries identified that paying for nutritional advice had benefits, including increased commitment to the service and motivation to follow the advice. Motivation is crucial for dietary behaviour change and is thought to be one of the most promising facets of personalising dietary advice^(^ ^7^ ^)^. Hence, the ironic consequence of free PN advice may be that it attracts more people, who are less motivated. The single act of having to pay for the advice may increase the motivation to adhere. If this is the case, providing PN advice at a cost may enhance its effectiveness compared with providing it for free of charge. The association of cheap/free PN with a poorer quality service and less likelihood of achieving benefits was also observed in primary analysis of Food4Me focus group data in the EU population^(^ ^33^ ^,^ ^34^ ^)^. Consideration of the relationship between cost and commitment should be a priority if provision of free PN advice is an intention in the UK.

As found previously^(^ ^33^ ^,^ ^34^ ^)^, the preferred method of PN delivery was face-to-face. Discussants perceived it easier to trust providers with personal information when communicating with them in person; it also gave them the opportunity to check the providers' professional credentials. In addition, there were concerns that the quality and quantity of feedback provided online would be less than that in a face-to-face consultation, and that it would take more time. Ease of use and accessibility has been identified as environmental factors probably to influence engagement in PN and, more importantly, behaviour change thereafter^(^ ^38^ ^)^.

In terms of providers of PN advice, the evidence suggests that being motivated by improving health outcome and benefiting the user, as opposed to profit, is crucial, as noted previously^(^ ^8^ ^,^ ^9^ ^)^. Additional factors that affected preference for PN provider were a good reputation, suitably qualified professionals and secure protection and storage of personal data. Privacy concerns regarding the storage and misuse of personal data, particularly genetic data, are well documented^(^ ^39^ ^,^ ^40^ ^)^, and it was perceived that these factors were more difficult to validate with online providers. Individuals presumed to be qualified to provide PN were dietitians, nutritionists and suitably qualified GP, who were perceived as the most trustworthy sources of nutritional advice^(^ ^41^ ^,^ ^42^ ^)^. Delivery of PN advice by a qualified expert or dietitian was also perceived as positive and highly appreciated in a previous analysis^(^ ^34^ ^)^. As identified in the primary analysis of Food4Me focus groups^(^ ^33^ ^,^ ^43^ ^)^, respondents perceived that PN should equate to a tailored service including physiological measurements and face-to-face consultation. However, there have been many developments with regard to Interactive Health Communication, and it is possible that the public are not aware of the potential to tailor information online, given that direct-to-consumer tests mainly operate through information delivery. It has been suggested that integrating Interactive Health Communication technologies into public health interventions is a more efficient way to utilise healthcare resources^(^ ^44^ ^)^, and there is a growing body of evidence that tailoring dietary information in this manner is efficacious in modifying dietary behaviours and reducing NCD risk^(^ ^45^ ^,^ ^46^ ^)^. A recent meta-analysis of online interventions for changing dietary and lifestyle behaviours has found added benefit of computer-based systems in addition to one-to-one contact, but not as a replacement method of delivery^(^ ^47^ ^)^.

Supplementary analysis of the Food4Me focus group data has enabled the in-depth exploration of the issue of the NHS on PN service delivery in the UK, which was only partially addressed in the primary analysis. The present study provided novel insight into preferences for PN delivery in the UK. It has also highlighted potential alternatives to face-to-face delivery by a HCP within the NHS, including referral to a private institution via an NHS HCP and the use of an online chat medium. Future research should focus on the wider acceptability of these methods of delivery, particularly if the NHS is unable to fund the provision of nutrigenomic-based PN.

It has been suggested that socio-economic status may influence perceptions and beliefs about PN^(^ ^48^ ^)^, although it is unclear how age, sex and knowledge may affect this^(^ ^25^ ^)^. Given the narrow socio-economic class of the participants in the present study, it was not possible to assess the impact of education on perceptions of PN service delivery. It was also not possible to detect any differences in opinion dependent on age or sex due to the low number of participants in each country. A further limitation is the mixed focus group design of the study, which can result in individual's opinions being influenced by other members of the group.

### Conclusion

The present supplementary analysis of focus group data, collected on behalf of the Food4Me study^(^ ^32^ ^)^, provides a novel insight into preferences for PN provider and delivery in the UK and Ireland. It is clear that participants in both countries prefer PN to be led by the government and for the costs for this to be equivalent to pre-existing healthcare services. However, the unique challenge of ‘free provision’ that is embedded in the NHS ‘culture’ adds complexities to its introduction in the UK, particularly given the association between paying for and commitment to a service. It is, thus, important that delivery of PN in the UK is carefully considered to facilitate its successful introduction into healthcare and to ensure that users are trustful of services offered. It is questionable whether the NHS could provide nutrigenomic-based PN as a preventative measure or whether consumers expect this. Referral by a HCP to a private healthcare company could be one possible alternative.
